# GlycCompSoft: Software for Automated Comparison of Low Molecular Weight Heparins Using Top-Down LC/MS Data

**DOI:** 10.1371/journal.pone.0167727

**Published:** 2016-12-12

**Authors:** Xiaohua Wang, Xinyue Liu, Lingyun Li, Fuming Zhang, Min Hu, Fuji Ren, Lianli Chi, Robert J. Linhardt

**Affiliations:** 1 Anhui Province Key Laboratory of Affective Computing and Advanced Intelligent Machine, School of Computer and Information, Hefei University of Technology, Hefei, China; 2 Center for Biotechnology and Interdisciplinary Studies, Rensselaer Polytechnic Institute, Troy, New York, United States of America; 3 National Glycoengineering Research Center, Shandong University, Jinan, China; 4 Wadsworth Center, New York State Department of Health, Albany, New York, United States of America; 5 Department of Information Science and Intelligent Systems, University of Tokushima, Tokushima, Japan; University of Patras, GREECE

## Abstract

Low molecular weight heparins are complex polycomponent drugs that have recently become amenable to top-down analysis using liquid chromatography-mass spectrometry. Even using open source deconvolution software, DeconTools, and automatic structural assignment software, GlycReSoft, the comparison of two or more low molecular weight heparins is extremely time-consuming, taking about a week for an expert analyst and provides no guarantee of accuracy. Efficient data processing tools are required to improve analysis. This study uses the programming language of Microsoft Excel^™^ *Visual Basic for Applications* to extend its standard functionality for macro functions and specific mathematical modules for mass spectrometric data processing. The program developed enables the comparison of top-down analytical glycomics data on two or more low molecular weight heparins. The current study describes a new program, GlycCompSoft, which has a low error rate with good time efficiency in the automatic processing of large data sets. The experimental results based on three lots of Lovenox^®^, Clexane^®^ and three generic enoxaparin samples show that the run time of GlycCompSoft decreases from 11 to 2 seconds when the data processed decreases from 18000 to 1500 rows.

## Introduction

Heparin is a complex, polydisperse and structurally heterogeneous mixture of linear, anionic polysaccharides that is widely used as a clinical anticoagulant [1]. Discovered 100 years ago in 1916, heparin’s use predated the establishment of the U.S. Food and Drug Administration (FDA) [1]. Low molecular weight (LMW) heparins, introduced in the 1990s for their improved pharmacodynamics and bioavailability, are derived from heparin by controlled depolymerization. LMW heparins, including innovator drugs and more recently generic versions, have undergone a great deal of regulatory scrutiny because of their polycomponent nature and their high level of structural complexity [2]. Studies of new approaches to determine the structure of LMW heparins have included bottom-up [3], top-down [4] and combined liquid chromatography (LC)-mass spectrometry (MS) analysis [5–6]. Despite these major advances, the rapid and accurate glycomic analysis of glycosaminoglycans by LC-MS has been plagued by the absence of suitable bioinformatics software.

In contrast to glycomic analysis, proteomic analysis using LC-MS is highly developed [7–8]. The availability of bioinformatics software has made routine proteomic analysis available to non-experts and has allowed expert laboratories to take on more difficult challenges, such as prost-translational modifications [9]. Recently, there has been an increased interest in developing similar bioinformatics software for glycomic analysis [10–11]. In the area of glycosaminoglycan analysis, GlycReSoft 1.0 software, developed at Boston University by Zaia and coworkers [12], relies on raw mass spectral data after auto-processed charge deconvolution using DeconTools software [13]. GlycReSoft has been applied to the top-down analysis of LMW heparins [4]. While this approach has been quite successful, the data coming from GlycReSoft for the comparison of the top-down of three lots of LMW heparin takes about one week of a skilled analyst’s time to manually process. Here we report the GlycCompSoft algorithm that allows the comparison of three sets of top down analysis from three different batches of LMW heparin in a few minutes.

## Materials and Methods

### Data preparation and pre-processing

Lovenox^®^ and Clexane^®^, the innovator versions of enoxaparin marketed in the U.S. and Europe were purchased from Sanofi-Aventis (Bridgewater, NJ). Generic versions of Lovenox^®^ were provided by three different manufactures (three current lots of each). Online hydrophilic interaction chromatography (HILIC) Fourier transform mass spectrometry (FTMS) was performed as previous described [4]. Briefly, enoxaparin injections were diluted into 1 μg/μL and directly injected into a HILIC column (2.0 mm × 150 mm, 200 Å, Phenomenex, Torrance, CA) by an Agilent 1200 autosampler. The LC column was directly connected online to the standard ESI source of LTQ-Orbitrap XL FT-MS (Thermo Fisher Scientific, San-Jose, CA). The enoxaparin intact chain compositions were analyzed under the negative mode. Following raw data acquisition, charge deconvolution was auto-processed using DeconTools [14–15] software. Enoxaparin structural assignment was performed by automatic processing with GlycReSoft 1.0 software, developed at Boston University (http://code.google.com/p/glycresoft/downloads/list) [4,12]. The output on enoxaparin composition from GlycReSoft was then processed using GlycCompSoft to provide automated relative quantification of intact chains present in enoxaparin.

### GlycReSoft parameters

Matching parameters were set as: Minimum Abundance, 1.0; Minimum Number of Scans, 1; Molecular Weight Lower Boundary, 500 Da; Molecular Weight Upper Boundary, 6000 Da; Mass Shift, ammonium; Match Error (E_M), 5.0 ppm; Grouping Error (E_G), 80 ppm; Adduct Tolerance (E_A), 5.0 ppm. The enoxaparin intact chain molecular weight hypothesis was generated by GlycReSoft [4]. Up to 18 saccharide units (degree of polymerization (dp)18) were analyzed. Hypothesis generating parameters were set as: A, ΔHexA = 0 or 1; B, HexA = 0−9; C, HexN = A + B − 1 to A + B + 1; D, Ac = 0−3; E, SO3 = B to A + B + (C*2) + 1 − D; Modification, Adduct = ammonium from 0 to 14. For the oligosaccharide with both 4,5-unsaturated NREs and 1,6-anhydro REs, there are two dehydration sites in one oligosaccharide molecule. “ΔHexA” was set as “1” and the molecular of “ΔHexA” was changed into C_6_H_4_O_4_ from C_6_H_6_O_5_ to add one more H_2_O loss. Other parameters were kept as the same. After matching the LC-MS raw data output by DeconTools [13] with the hypothesis, GlycReSoft gives out 15 features [12]. GlycCompSoft then compares and screens the full scale matching results output by GlycReSoft based on *Compound Key*, *Total Volume*, *Scores* and other features automatically.

### GlycCompSoft algorithm

**Fig 1:**
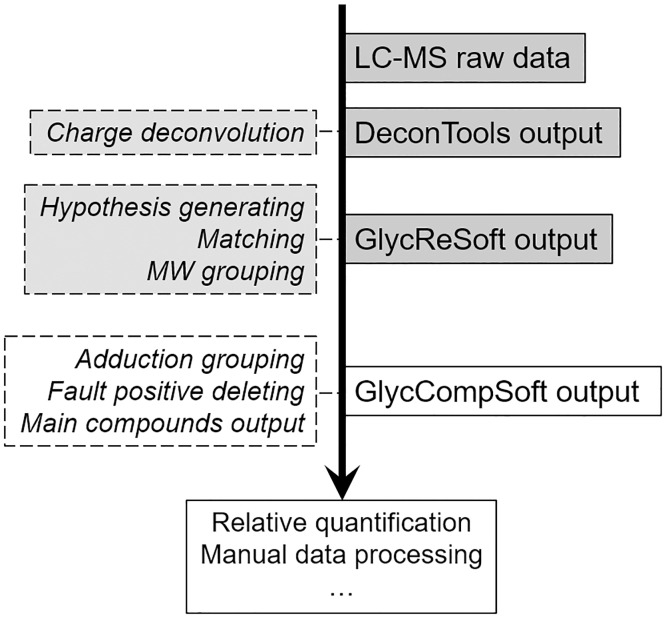
Total workflow of enoxaparin intact chain LC-MS analysis coupling with software processing.

**Fig 2:**
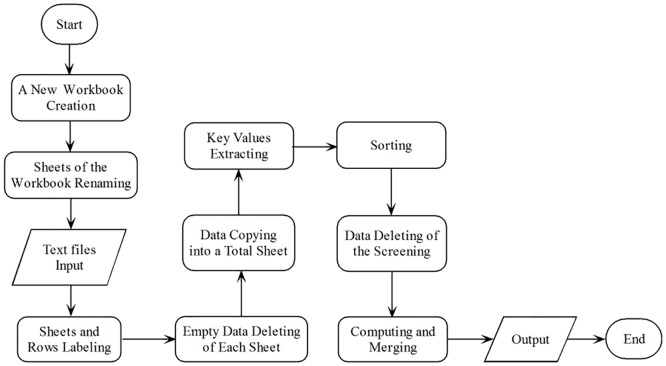
The Overall Flowchart of the GlycCompSoft from text file(.txt) input to true glycans data screened and merged.

The workflow from LC-MS to relative quantification is shown in Fig 1. GlycCompSoft compares and screens the full scale matching results output by GlycReSoft. Matching results of three batches/replications output by GlycReSoft can be input into Microsoft Excel in text format (.txt) and can be automatically compared, screened and computed by GlycCompSoft. First, a new workbook is created and data from three text files are input into three sheets of the workbook. Each sheet is renamed using the text file name and a new column is also added to each sheet with the value of the text file name, so that each row of the output can be labeled and distinguished based on where it comes from in the comparison and screening process. Second, all data in each sheet with the empty value in the Compound Key feature [12] is deleted to allow true signals arising from glycosaminoglycan chains to be distinguished from noise and to make GlycCompSoft more efficient all the rows in the three sheets are copied to a new sheet named with “Total Sheet”. Third, data in the Total Sheet is sorted, compared, screened, computed and merged. The overall flowchart of the GlycCompSoft presented in Fig 2. A full copy of the program and the user guide are presented in S1 File.

**Fig 3:**
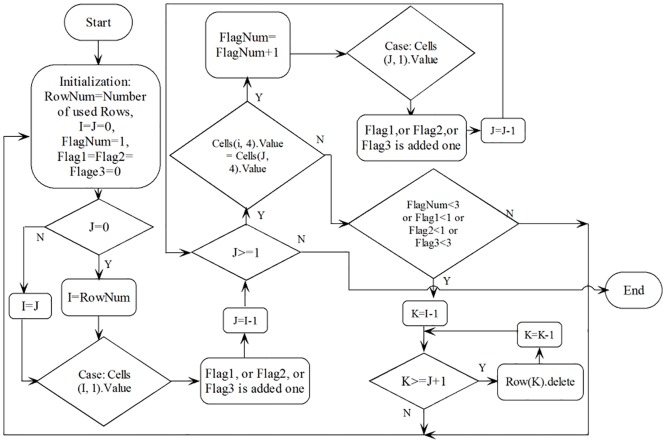
Flowchart of the Deletion Module for screening of the GlycCompSoft.

In the third step, the value of Compound Key feature in each row is first extracted. The entire or partial list of Compound Key values, enclosed with square brackets in each cell, is extracted according to the lazy matching method in regex [16–18], which instructs the engine to match as few input characters as possible and then proceed to the next token in the regular expression pattern, improving the efficiency of value matching. In order to compare and check each row of data in the “Total Sheet” accurately and efficiently, all the rows are sorted according to the extraction value of Compound Key feature. If at least three rows of data have the same extraction values of the Compound Key column, and if these three or more than three rows of data come from three different sheets (text files) and can be distinguished by the newly added column with the value of the sheet (text files) name, then these rows of data are retained, otherwise they are deleted as noise. The flowchart of the deletion module is shown in Fig 3. For the retained data, when two or more have same extracted Compound Key value rows will be merged as the row with the largest value in the Score feature will be retained and the sum values of Total Volume will be calculated and adopted, the algorithm of the merging module is shown in Table 1.

**Table 1:** Merging procedure Module Algorithm of GlycCompSoft.

Algorithm of Merging
Initial I using the number of the Used Range rows
Sort all the data according to the Compound Key feature
For J = I−1 to 1
If Value of Cells (I, Compound Key) = Value of Cells (J, Compound Key)
And Label Value of Cells (I) = Label Value of Cells (J)
Then
If Value of Cells (I, Score) ≤ Value of Cells(J, Score)
Then
Value of Cells (J, Total Volume) = Value of Cells (J, Total Volume) + Value of Cells (I, Total Volume)
Delete Rows(I)
I = J
Else
Value of Cells (I, Total Volume) = Value of Cells (I, Total Volume) + Value of Cells (J, Total Volume)
Delete Rows(J)
I = J
End If
Else: I = J
End If
Next J

## Results and Discussion

Enoxaparin is the most widely used low molecular weight (LMW) heparin and is derived from heparin through alkaline β-eliminate cleavage [2]. Heparin is polydisperse with its major repeating unit, [→4) IdoA2S (1→4) GlcNS6S (1→]_n_, comprising 60–90% of its structure [2,19]. In addition, there are a number of undersulfated residues, including IdoA, GlcNS, GlcNAc6S GlcNAc and GlcA, which are present in much lower amounts. Both heparin and LMW heparin also contain an active site called the antithrombin-binding pentasaccharide. These sites can have variable structures, however, they always contain a central Glc(NAc/NS)(6S/OH)3S residue. The intact chain composition in enoxaparin (average molecular weight (Mw) ~ 4.5 kDa) is more complicated than its heparin precursor Mw ~ 16 kDa) since the controlled cleavage of heparin through chemical β-elimination generates 4,5-anhydro uronic acid, ΔUA2S and ΔUA, at the non-reducing ends (NREs) of the LMW heparin product chains. Also a fraction (~16%) of the LMW heparin chain reducing ends (REs) are1,6-anhydro hexosamines. More than 120 structural compositions have been reported enoxaparin oligosaccharide chains [4–5].

The intact chain composition of enoxaparin after LC-MS has been successfully analyzed using GlycReSoft [4]. In this analysis, a total HILIC-FTMS time of 70 min resulted in more than 15,000 components that were first summarized by DeconTools software to obtain acceptable resolution. After matching with the enoxaparin hypothesis, generated by GlycReSoft 1.0, on average about 450 components were obtained for each sample. Since the intact chain composition is complicated and the NH_3_ adduction varies, false positives matching results were present in almost 50% of the components. GlycReSoft provides a set of features and summarizes the features into a single score [12], which can determine false positives. However, for LMW heparin data, the score system usually also results in false negatives after manual confirmation (see S2 Table, where the true positive result [8,8,1,12,0] has a score similar to the false positive result [8,8,1,14,1]). The best way to maximize the number of likely components is retain all the components that are found in three replicate determinations or on three lots of the same drug based on the “All-presence-principle”. The reliability of this “all-presence-principle” approach can be confirmed by manually checking the most abundant oligosaccharides. In the absence of an automatic approach, one can only manually keep components present in the three replicates, a time-consuming process with a high error rate. The workflow for determining the relative quantification of enoxaparin intact chain composition from LC-MS is shown in Fig 1. The workflow and illustration of GlycCompSoft, which is designed based on this process is shown in Table 2.

**Table 2:** The overall workflow and illustration of GlycCompSoft.

	Step name	Illustration
1	Input Matching Result	Copy supervised GlycReSoft matching result into three blank excel sheet provided by GlycCompSoft
2	Name Data Sets	-
3	Remove Non-glycan Data	Delete rows with empty GlycReSoft matching columns
4	Sample Names Labeling	Label Glycan data with original sample names in a new row before all data to make sure the data could be extracted after matching
5	Mix all Three Lots into a NewSheet for Comparing	Insert a total comparison sheet and copy data of three lots/replications into the new sheet
6	Sort by Macthing Results	To check if a compound is present in three lots/replications
7	Extract Compound Names	To get rid of the NH3 adduction difference GlycReSoft gives out
8	Delete False Positive Results	Delete compounds without fully presence in three lots/replications
9	Adductiong Grouping	Sum compounds with same compound names generated from step 8
10	All in One	Output of the analysis

The GlycCompSoft workflow is designed based on a manual process procedure.

### Experimental design

**Fig 4:**
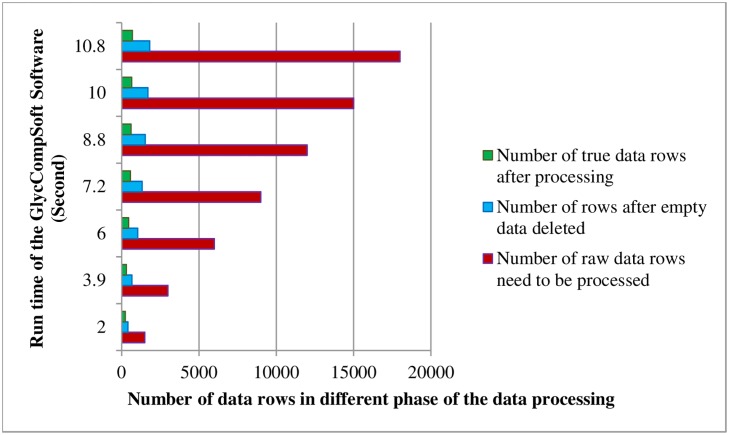
The runtime of the GlycCompSoft, and the comparison between rows of raw data, half-processed data and final data.

**Fig 5:**
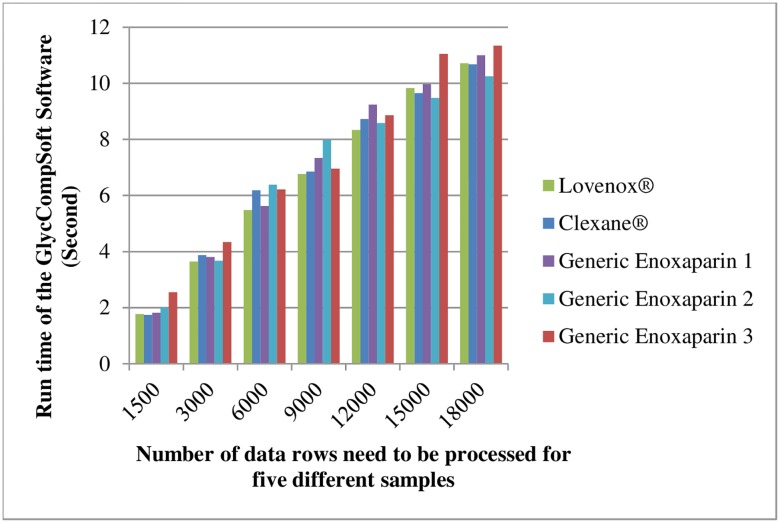
Time efficiency of GlycCompSoft based on Lovenox^®^, Clexane^®^, and generic enoxaparin 1,2 and 3 with different amounts of data rows.

A large number of experiments were on the Windows 8 Professional Operating System (Intel i7 2.1GHz, 8G RAM) to verify the accuracy and efficiency of the GlycCompSoft. Matching results for three lots of Lovenox^®^, Clexane^®^ and generic enoxaparins were used in the verification of this software. The proportion of the data rows without empty Compound Key feature values represents a relatively small portion of the entire data set derived from the data files. However, the proportion of the true data finally filtered is much lower. The detailed information is shown in Fig 4, and the red, blue and green bars represent the amounts of raw data, the data after the rows with empty Compound Key are deleted, and the true data, respectively. Thus, the challenge is to have a low error rate with good time efficiency in comparing, screening and computing these massive data sets by a manual method even though the sorting procedure can be done using Excel [20]. While ensuring a low error rate, GlycCompSoft can automatically perform this process within several hundred seconds. It is noteworthy that the runtime can be further reduced to ten seconds by deleting the empty value rows before copying to the Total Sheet (Table 3). Thus, it is important to delete the rows with empty values in Compound Key feature before copying data to the Total Sheet. The runtime of the GlycCompSoft decreases with the decreasing numbers of data rows requiring processing and at 18000 rows, the average runtime is under 11 seconds (Fig 5 and Table 3). Detailed runtimes for the five different samples at a different number of rows are shown in Fig 5.

**Table 3:** Performance of the GlycCompSoft.

Number of raw data rows			1500	3000	6000	9000	12000	15000	18000
**Number of true data rows**			241	301	450	571	611	652	695
**Screening accuracy**	Manual processing		>95 in average and varies on analysts						
	GlycCompSoft		100	100	100	100	100	100	100
**Runtime**	Manual processing		From several hours to 1–2 workdays						
	GlycCompSoft	Keep empty matching results and compare	39.9	85.9	175.4	251.9	348.3	467.3	521.4
		Delete empty matching results first and compare	2	3.9	6	7.2	8.8	10	10.8

### Enoxaparin comparison results

**Fig 6:**
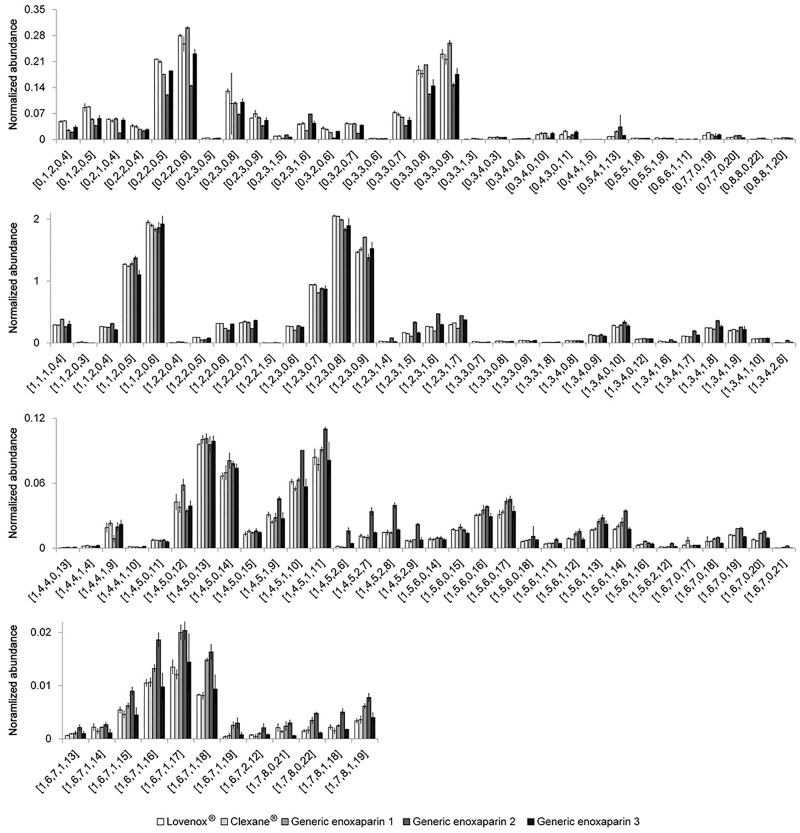
Relatively quantitative comparison of identified enoxaparin oligosaccharides without 1,6-anhydro reducing end from five commercialized LMW heparin products. Oligosaccharide compositions are given as [ΔHexA = 0 or 1, HexA, GlcN, Ac, SO_3_].

**Fig 7:**
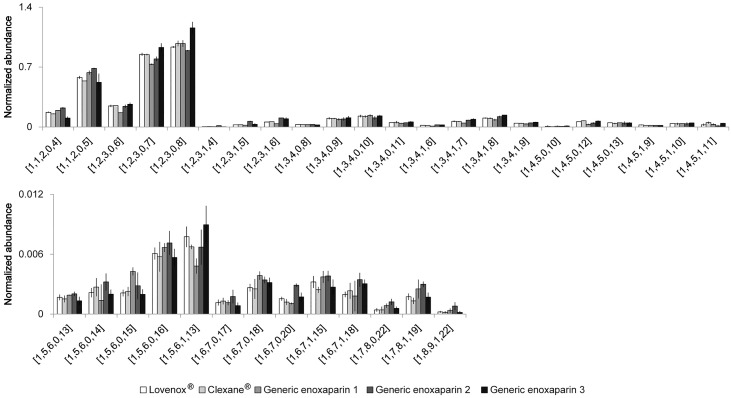
Relative quantitative comparison of identified enoxaparin oligosaccharides with 1,6-anhydro reducing end from five commercialized LMW heparin products. Oligosaccharide compositions are given as [ΔHexA = 1, HexA, GlcN, Ac, SO_3_].

Two enoxaparin innovator drugs and three generic drugs provided by different manufactures were compared. The abundance of each component was normalized to the sum of the total volumes of the 10 most abundant components. The major components (oligosaccharide compositions are given as [ΔHexA = 1, HexA, GlcN, Ac, SO_3_] [12]) are shown in Fig 6. The innovator drugs, Lovenox^®^ and Clexane^®^, are believed to be prepared through the same manufacturing process and their major components give an almost identical analysis. Generic enoxaparin-1 shows some subtle differences when compared to the innovator drugs and somewhat greater differences are seen in generic enoxaparin-2. Generic enoxaparin-2 contained greater amounts of *N*-acetyl substituted chains than the other LMWHs, as can be clearly observed in the dp10 components ([1,4,5,2,7]-[1,4,5,2,9]). Enoxaparin-1 shows a similar *N*-acetyl trend as Enoxaparin-2 in the dp12 components ([1,5,6,0,14]-[1,5,6,2,12]) and in the dp14 components. Chains with saturated NREs, derived from the original NRE of the parent heparin (oligosaccharide compositions are given as [ΔHexA = 1, HexA, GlcN, Ac, SO_3_]), are shown in Fig 6. Enoxaparin-2 was lower in these chains, suggesting it was derived from a heparin precursor of a higher molecular weight. Finally, oligosaccharides with 1,6-anhydro RE sugars, coming from a side reaction in the process chemistry (oligosaccharide compositions are given as [ΔHexA = 1, HexA, GlcN, Ac, SO_3_]), are shown in Fig 7. A higher *N*-acetyl in enoxaparin-2 was also observed in these chains (dp6 [1,2,3,1,4]-[1,2,3,1,6]).

### False discovery rate

GlycCompSoft is aimed at comparing complicated oligosaccharide mixtures. The false rate of the software calculation is technically 0%, while the false rate of oligosaccharide recognition is instead associated with the GlycReSoft software [12]. GlycReSoft has a many parameters that can be optimized, such as the minimum matching abundance and error. Moreover, GlycReSoft provides a score system to avoid false discovery as much as possible. However, for very complicated oligosaccharide mixtures, such as enoxaparin, the scoring system is not 100% reliable. Based on our manual interpretation experience (data not shown), the score threshold suggested by GlycReSoft, 0.16 in the software application on HS samples, is too high for the low abundant oligosaccharides in enoxaparin. Thus, in data analysis we first reduced the score threshold into 0.1, then used the GlycCompSoft to compare the oligosaccharide matching result output by GlycReSoft using our “all-presence-principle” before we used GlycReSoft score threshold filtration. The false discovery rate (FDR) of the total process is basically the FDR after our “all-presence-principle” associated with the GlycReSoft score system. Calculating an accuracy for the FDR based on enoxaparin oligosaccharide composition analysis is challenging because of the compositional complications of chain length, substitution, and diffuse abundance ions.

Therefore, the FDR was instead tested using the same LC-MS system and software processing by investigating four homogeneous chemoenzymatically synthesized heparin oligosaccharides (A-D) generously provided by Professor Jian Liu at the University of North Carolina. The structures of the oligosaccharides are presented in S1 Fig, and their full mass spectra are shown in S2 Fig. The number of GlycReSoft matching results from the LC-MS data of oligosaccharide (A) was 23, 18, 19, respectively, for 3 replicates. After filtering with GlycCompSoft, the number of results was reduced to 5 in each replicate. Then we performed a manual confirmation after which we found that only 3 of these were truly positive. Based on oligosaccharide (A), the false positive rate after GlycCompSoft processing was 40%, which is greatly decreased from an average false positive rate of 85% using only GlycReSoft matching. Similarly, by using GlycCompSoft the false positive rates for oligosaccharides (B, C and D), decreased from 89% to 62.5%, 87% to 50%, and 89% to 40%, respectively. Detailed GlycCompSoft comparison results are presented in S1, S2, S3 and S4 Tables. Since all expected oligosaccharides as well as unexpected contaminants were observed, no false negatives occurred during the software automatic processing.

### GlycCompSoft applications on other complicated carbohydrate mixtures

The capability of extracting useful information from a large LC-MS data set from LMW heparins has been demonstrated in the current study. GlycCompSoft is a powerful tool when used together with DeconTools and GlycReSoft on heterogeneous glycan products, including both intact chain compositions (as demonstrated here) and potentially on compositions of enzymatic of chemical cleavage components. Data can be processed in a short time as long as good LC-MS resolution is obtained. Different saccharide compositions (*i*.*e*., polysaccharides containing galactosamine, galacturonic acid, fucose, etc.,), different glycosidic bonds (1→2, 3, or 4) or the presence of side chains or branches do not present difficulties for GlycReSoft, as matching only relies on accurate molecular weight data. Based on the “All-presence-principle”, the extraction results in GlycCompSoft output that is highly accurate on high abundant components. While, automated data processing software, such as DeconTools, GlycReSoft, and GlycCompSoft, greatly reduce the data processing times, sometimes curation by a skilled analyst is required. For example, in cases where low abundance components are present only in some of the batches manual examination of the data may be required.

## Conclusions

GlycCompSoft is an algorithm that automates the comparison of complex data sets generated in the top-down analysis of LMW heparins. An “all-presence-principle”, makes GlycCompSoft a highly accurate method for the analysis of components present in high and moderate abundance. Automated data processing software, such as DeconTools, GlycReSoft, and GlycCompSoft, improve the speed and reliability of interpreting these complex data sets. Manual curation by a skilled analyst is still required in cases where low abundance components are selectively present only in some of batches of LMW heparins.

GlycCompSoft utilizes macro functions and specific mathematical modules in programming for massive data sets comparison, screening and computing. Work is ongoing that will include additional algorithms and error calculation methods to optimize the performance of this software. Ultimately machine learning methods should be applied that can accept training data from inputs and generate more intelligent output results based on different samples and relationships among their features. Future studies are also planned to examine the automated processing of LC-MS/MS data when reliable data sets become available.

## Supporting Information

S1 Fig Structures of the synthesized oligosaccharides. (TIF)

S2 Fig Mass spectra of the synthesized oligosaccharide. (TIF)

S3 Fig Mass spectra of the hexassacharides shown in S5 Table. (TIF)

S1 File User guide for GlycCompSoft program running. (PDF)

S2 File GlycCompSoft Software. This software can also be downloaded at www-heparin.rpi.edu. (XLSM)

S3 File GlycReSoft matching result for false discovery rate investigation. (XLSX)

S1 Table Components outputted by GlycCompSoft from synthesized oligosaccharide (A). Components are given out as [HexA, GlcN, PNP = 1, SO3, Ac], and results in red were confirmed as false positive results after manual interpretation.
(DOCX)

S2 Table Components outputted by GlycCompSoft from synthesized oligosaccharide (B). Components are given out as [HexA, GlcN, PNP = 1, SO3, Ac], and results in red were confirmed as false positive results after manually interpretation.
(DOCX)

S3 Table Components outputted by GlycCompSoft from synthesized oligosaccharide (C). Components are given out as [HexA, GlcN, PNP = 1, SO3, Ac], and results in red were confirmed as false positive results after manually interpretation.
(DOCX)

S4 Table Components outputted by GlycCompSoft from synthesized oligosaccharide (D). Components are given out as [HexA, GlcN, PNP = 1, SO3, Ac], and results in red were is confirmed as false positive results after manually interpretation.
(DOCX)

S5 Table Four kinds of sulfated hexasaccharides detected in the three kinds of generic enoxaparin. After matching the experimental data with the hypothesis generated by GlycoReSoft, the matching results were further filtrated by GlycoCompSoft using all-presence-principle. The table was outputted as the final matching result. (Components are given out as [ΔHexA = 1, HexA, GlcN, Ac, SO3]).
(DOCX)
