# Supplementary figures and images for: GlycCompSoft: Software for Automated Comparison of Low Molecular Weight Heparins Using Top-Down LC/MS Data
Source: PLoS One. 2016 Dec 12;11(12):e0167727. doi: 10.1371/journal.pone.0167727 (PMC5152843; doi:10.1371/journal.pone.0167727)

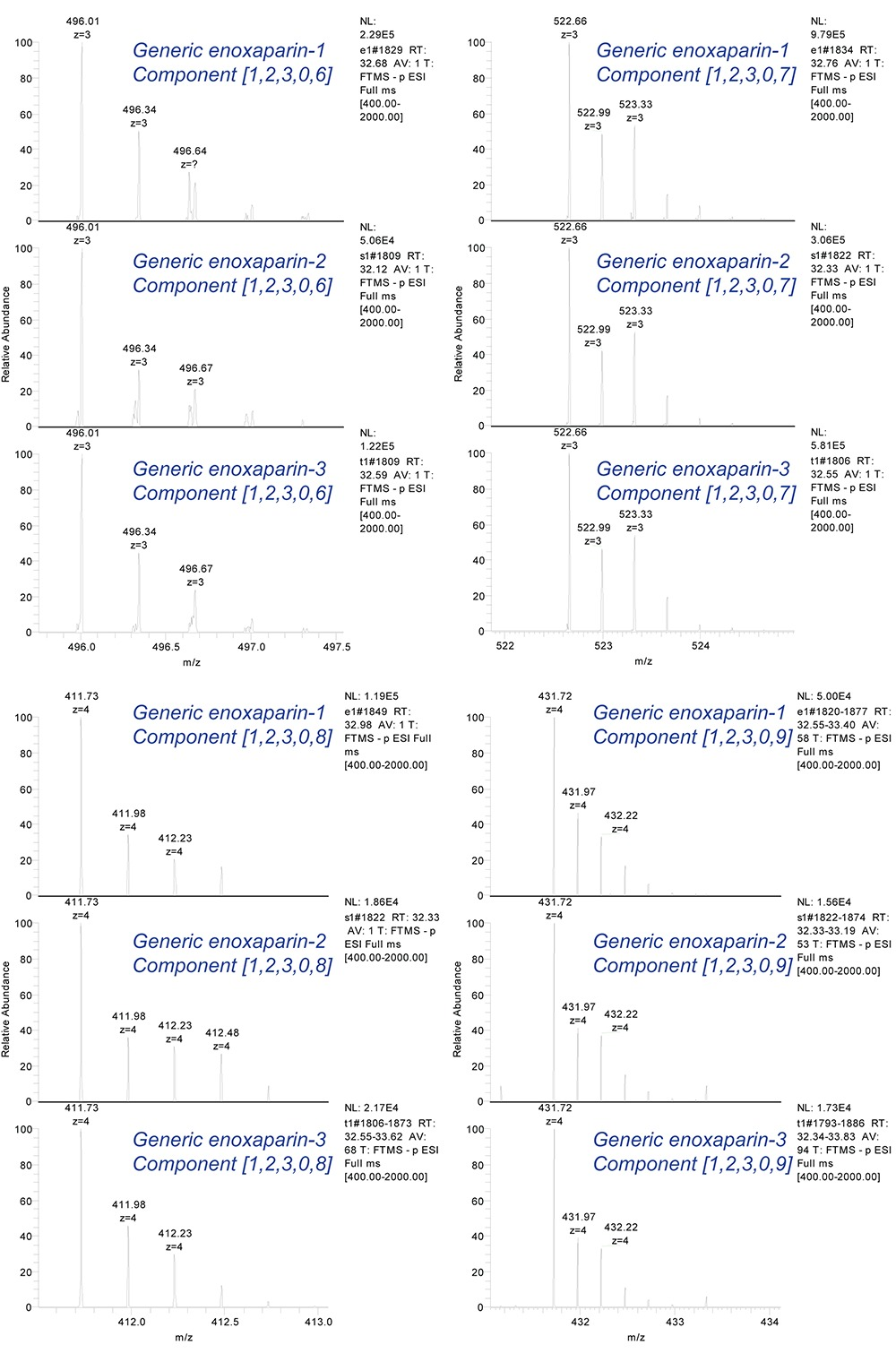

Supplement: S1 Fig — (TIF) [file pone.0167727.s001.tif]

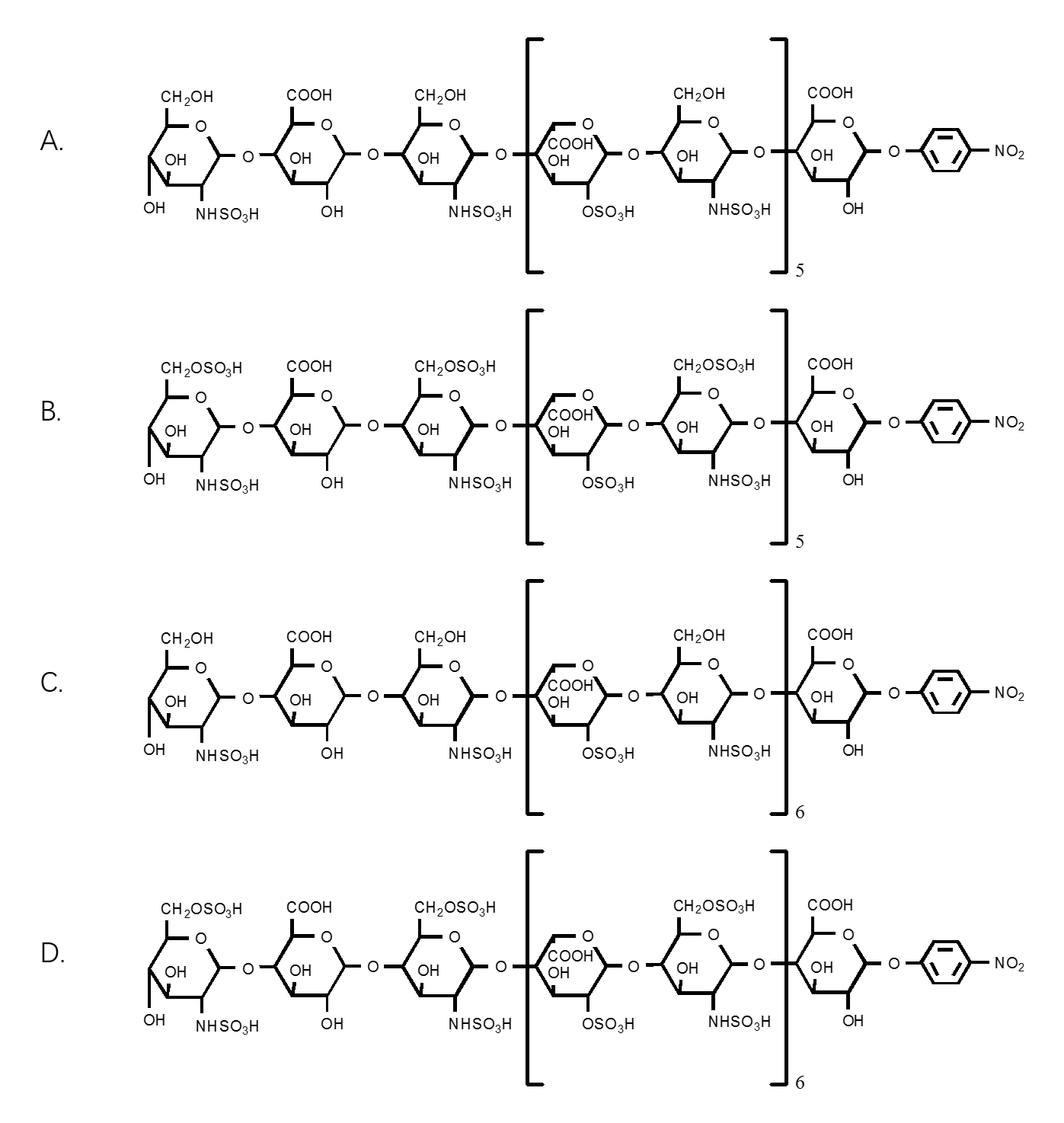

Supplement: S2 Fig — (TIF) [file pone.0167727.s002.tif]

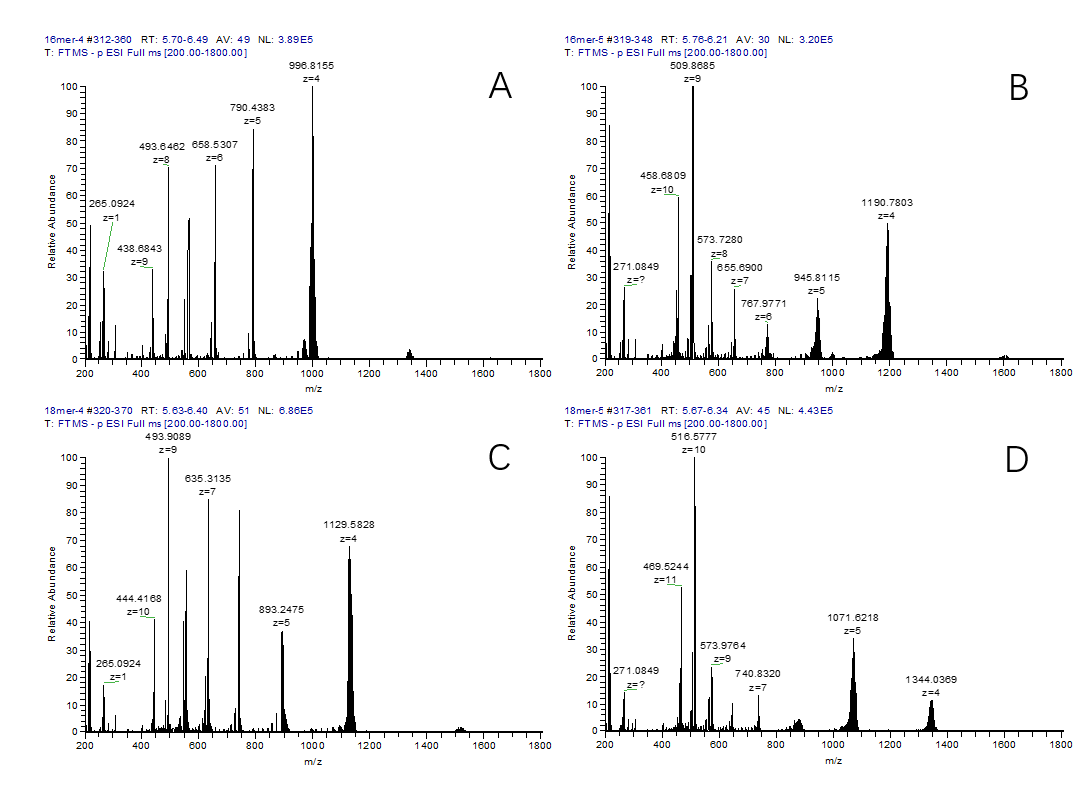

Supplement: S3 Fig — (TIF) [file pone.0167727.s003.tif]
