# Supplementary material for: GlycCompSoft: Software for Automated Comparison of Low Molecular Weight Heparins Using Top-Down LC/MS Data
Source: PLoS One. 2016 Dec 12;11(12):e0167727. doi: 10.1371/journal.pone.0167727 (PMC5152843; doi:10.1371/journal.pone.0167727)
Supplement: S1 File — (PDF) [file pone.0167727.s004.pdf]

User Guide for the program GlycCompSoft

1. Program running. Double click the file of the GlycCompSoft.xlsm. The Excel is open and the window "Control Window of the GlycCompSoft" appears. The interface of the program is shown in Fig.1.

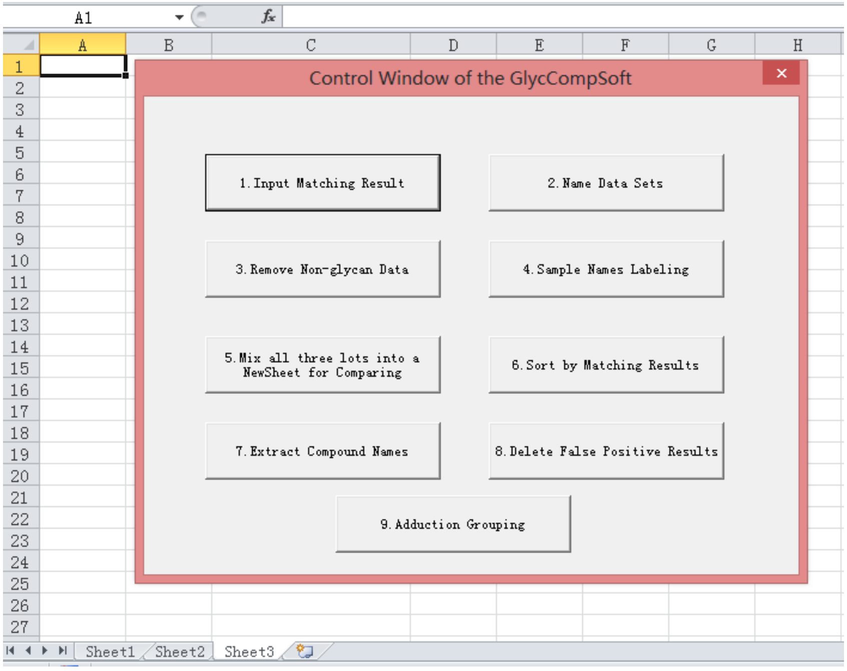

Fig.1 Program running interface

2. Input (or paste) matching result into the Excel Sheets. For the matching result perhaps come from different Softwares, has different structures and includes all kinds of separate characters, so input or paste data into sheets directly is a convenient method. The data will be input from Cell (1,1) of the three sheets (As shown in Fig. 2).

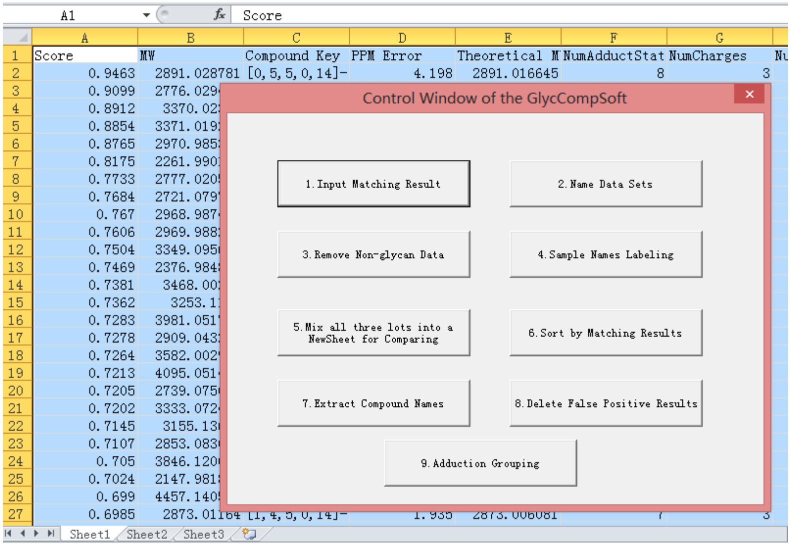

Fig.2 Data inputting

3. The sheets will be renamed with the corresponding text file names after clicking of the second button. That is to say the labeled sheets' names can be used to distinguish where data comes from in the comparison and screening process. The effect is shown in Fig.3.

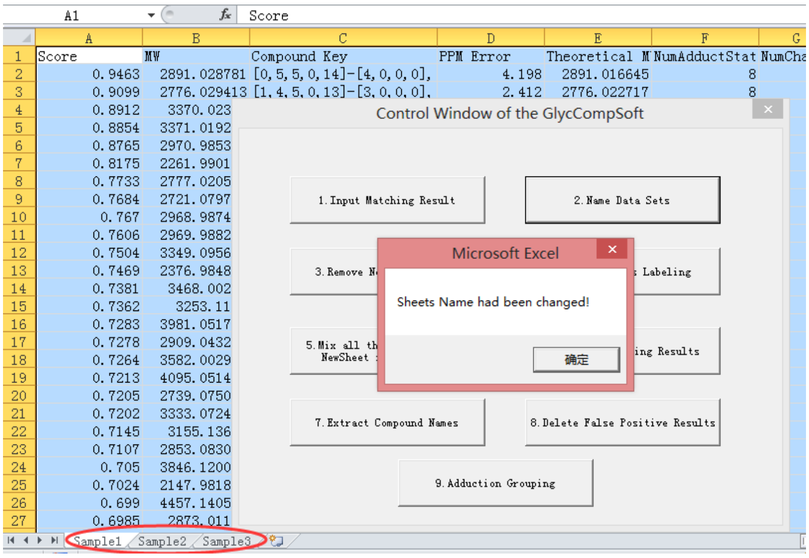

Fig.3 Sheets name changing

4. Click the third button to delete the rows with special values (for example, empty values), so as to remove non-glycan data from the sheets. And the original number of rows before and after deleting will be counted (as shown in Fig.4 and Fig.5 respectively).

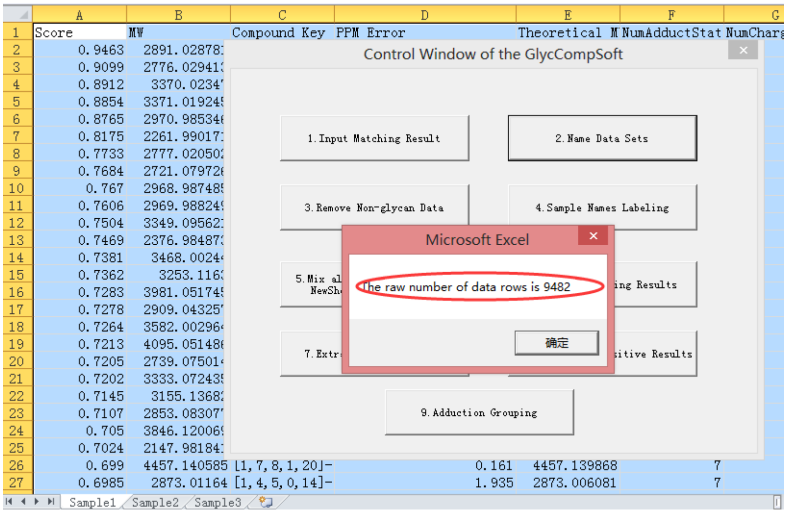

Fig.4 Raw number of data rows counting

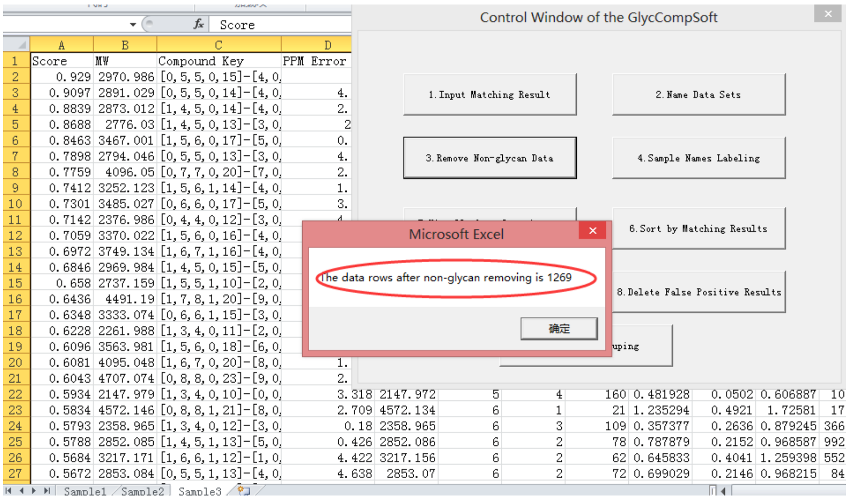

Fig.5 Data rows counting after non-glycan removing

5. Click the fourth button can get the effects including add a new column, label values for each new cell with the value of the name of each sheet, and color each sheet in three different colors(as shown in Fig.6). So each row of data can be distinguished where it comes from by the value of the first column or its background color in the following process.

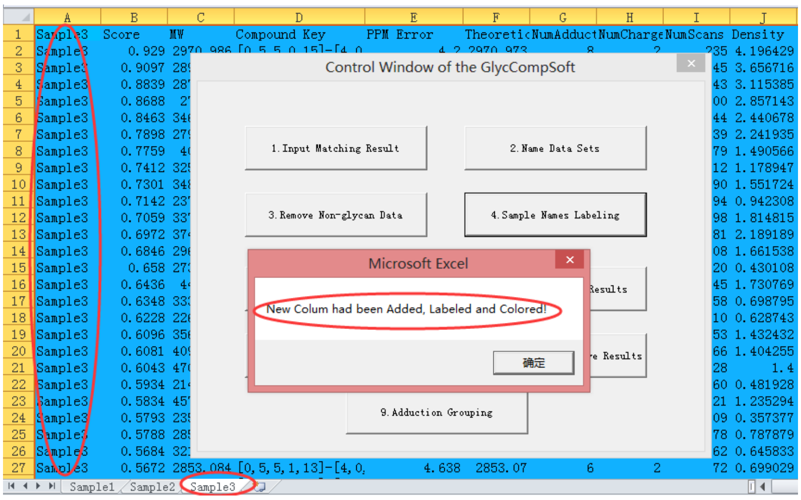

Fig.6 The Effects of the fourth button

6. For the following comparison and screening process, a new sheet is added to deposit data of the three sheets. The effect of the fifth button is shown in Fig.7, all data is gathered together in the new “Total Sheet” with different background colors.

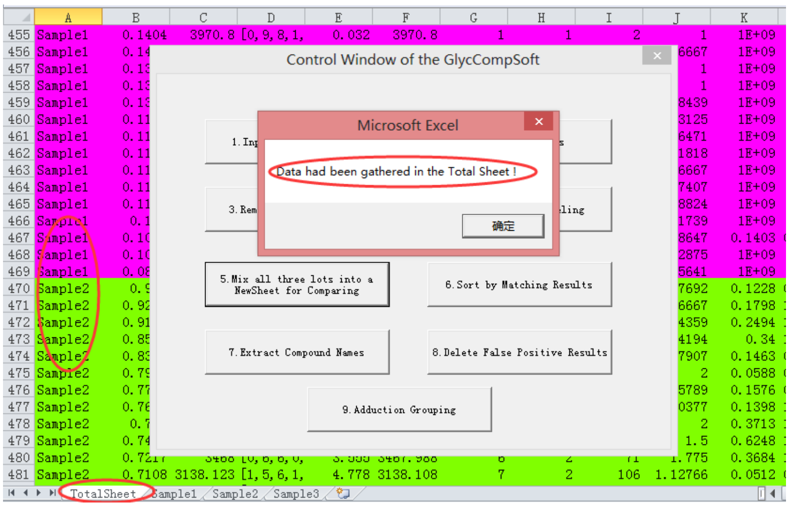

Fig.7 The effect of the fifth button

7. All the data in the "Total sheet" is sorted by matching results (the values of column D/Compound Key Value). The effect of the sixth button is shown in Fig.8.

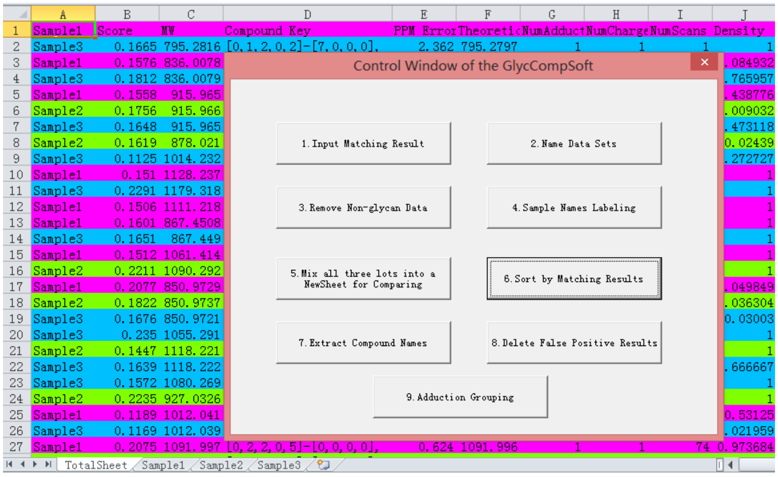

Fig.8 The effect of the sixth(Sorting) button

8. Only the first part values in the column D is necessary, so the first part (Compound Names) are extracted by the seventh button. The effect of the seventh button is shown in Fig.9

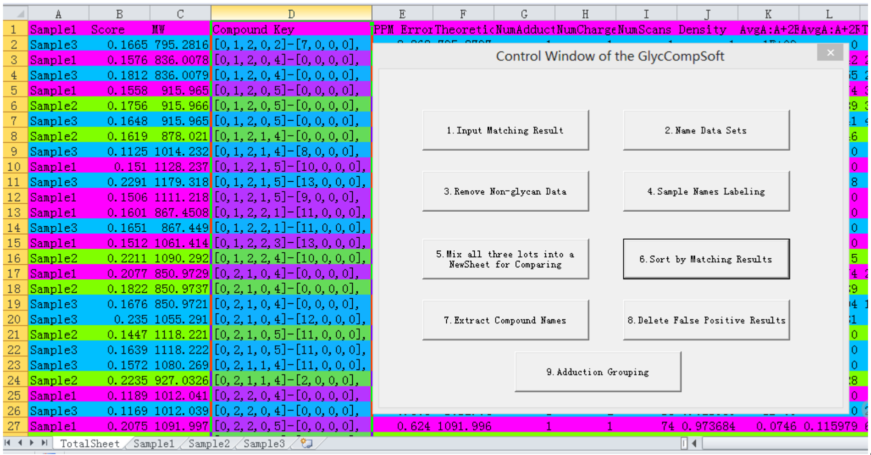

.

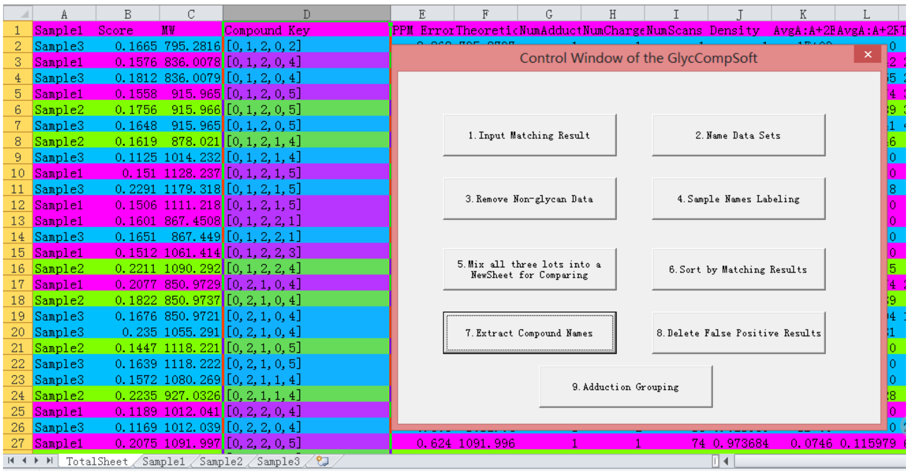

Fig.9 The effect of the Extracting button

9. False positive results in Total Sheet need to be deleted. In the GlycCompSoft program, data that could not meet the “All-presence-principle” defined in the manuscript can be compared and screened based on the values of column A and D. The data and row number before and after false positive results deleting are offered. The effects of the button eight are shown in Fig.10 and Fig.11.

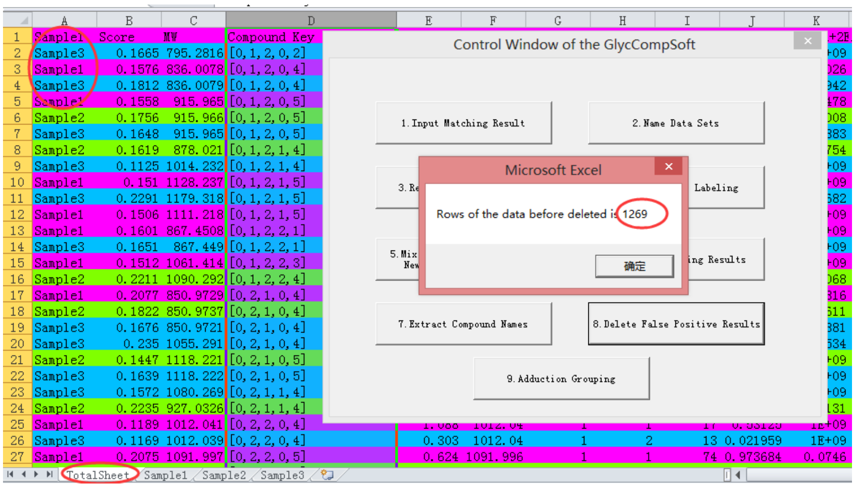

Fig.10 Data and row number shown before false positive data deleting

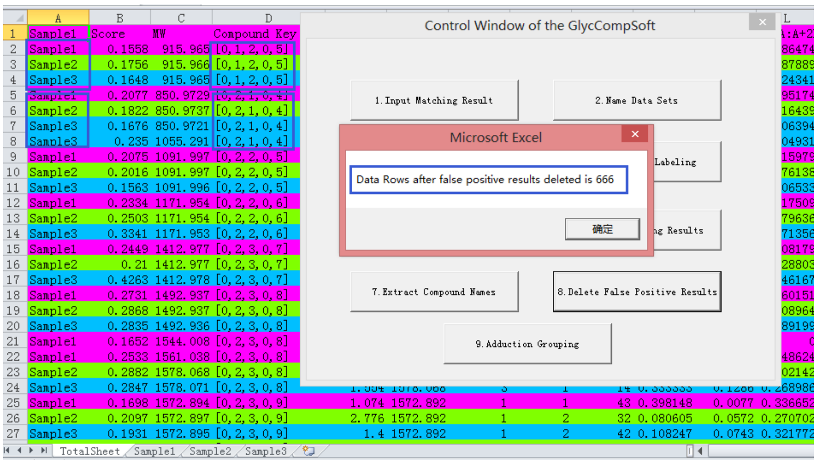

Fig.11 Data and row number shown after false positive data deleting

10. At this step the operation is adduction grouping. The reserved data from last step can be merged based on values of column A, B, D and M. The data and row number after merging is offered. The effect of the button labeled Adduction grouping is shown in Fig.12.

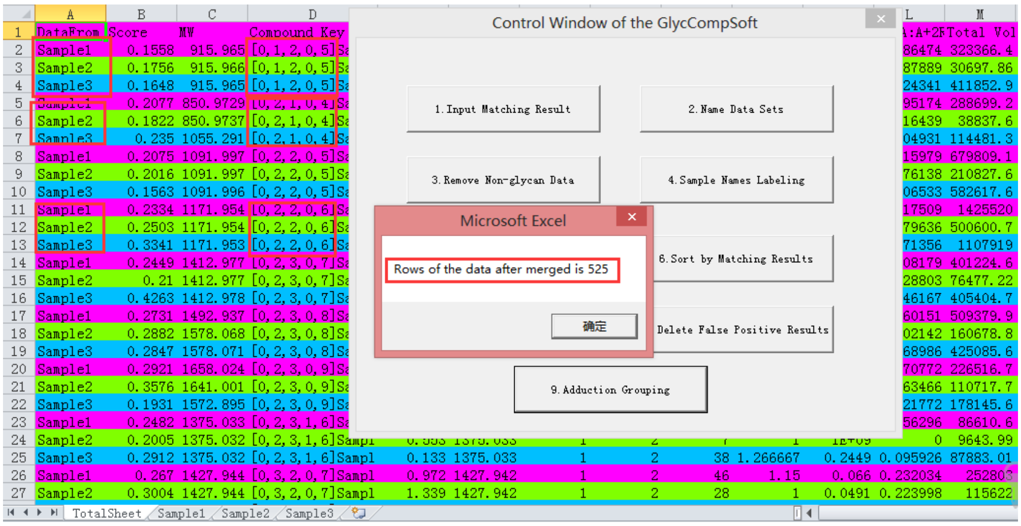

Fig.12 The effect of the button labeled Adduction grouping

11. The sheet named "Total Sheet" and the data in other sheets can be deleted (as shown in Fig.13). And the program can be run repeatedly.

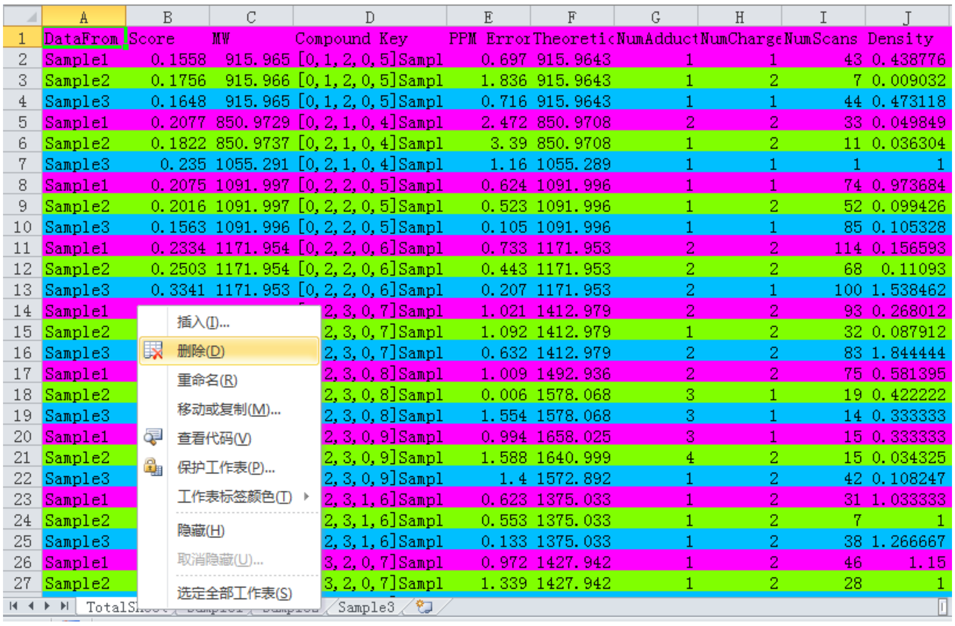

Fig.13 The Sheets can be emptied and rerun

12. The effect of Work Book and Work Sheets after data deleting is shown in Fig.14 and ready to rerun. The sheets names can be reserved or changed according to users' demands.

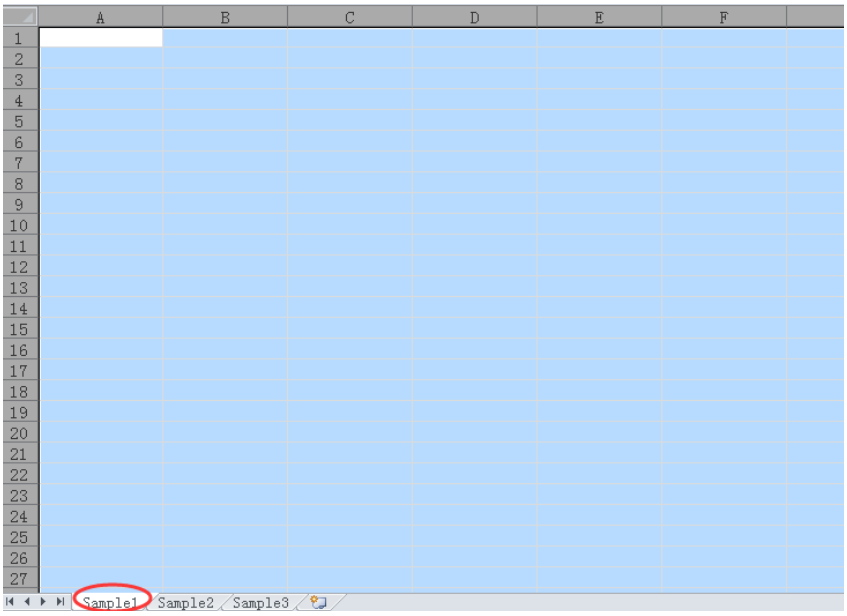

Fig.14 The Work Book and Work Sheets can be recovered and ready to rerun

The step to step method above could help users to observe data in different phase. If users only interested in the final results, all the steps can be combined together and the whole process will run automatically by the "All in one" button(Fig.15)

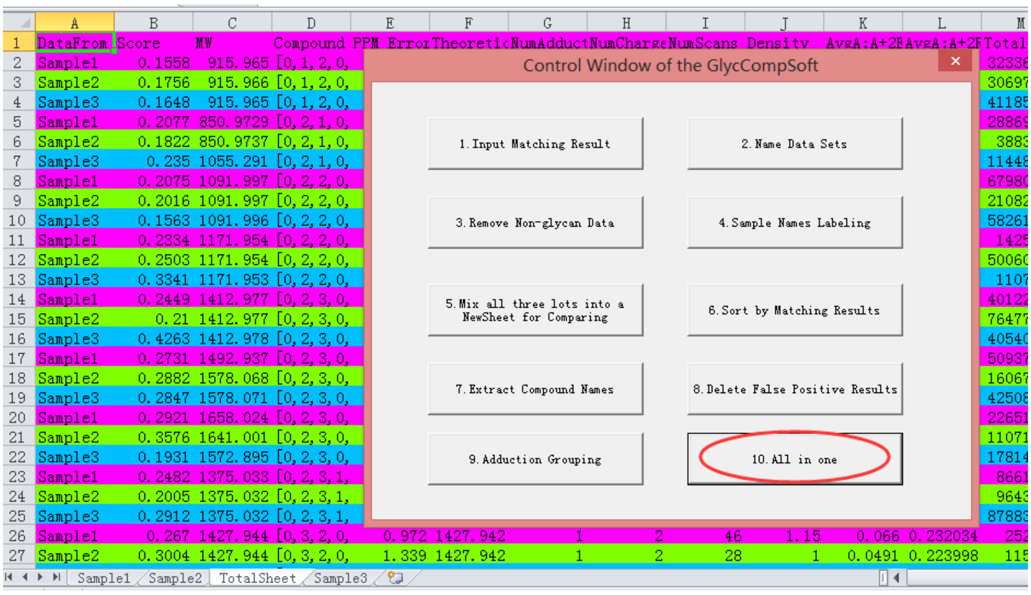

Fig.15 The whole process can be operated by the button of nuber 10 automatically

This user guide is submitted together with the following revised manuscript. Title of the manuscript is "GlycCompSoft: Software for automated comparison of low molecular weight heparins using top-down LC/MS data".

OCT. 28,2016
