# Supplementary material for: GlycCompSoft: Software for Automated Comparison of Low Molecular Weight Heparins Using Top-Down LC/MS Data
Source: PLoS One. 2016 Dec 12;11(12):e0167727. doi: 10.1371/journal.pone.0167727 (PMC5152843; doi:10.1371/journal.pone.0167727)
Supplement: S1 Table — Components are given out as [HexA, GlcN, PNP = 1, SO3, Ac], and results in red were confirmed as false positive results after manual interpretation. (DOCX) [file pone.0167727.s007.docx]

S1 Table

| DataFrom | Score | MW | Compound Key | PPM Error | Theoretical MW | NumCharges | Total Volume |
| --- | --- | --- | --- | --- | --- | --- | --- |
| Replicate 1 | 0.16 | 3795.3047 | [8,8,1,12,0] | 2.96 | 3795.3159 | 6 | 887188 |
|  | 0.31 | 3875.2814 | [8,8,1,13,0] | 2.25 | 3875.2727 | 6 | 9392888 |
|  | 0.89 | 3955.2390 | [8,8,1,14,0] | 2.40 | 3955.2295 | 7 | 65797412 |
|  | 0.16 | 4031.2882 | [8,8,1,14,1] | 1.23 | 4031.2932 | 2 | 2039888 |
|  | 0.13 | 3482.1932 | [9,9,1,1,1] | 0.34 | 3482.1944 | 2 | 16567 |
|  |  |  |  |  |  |  |  |
| Replicate 2 | 0.18 | 3795.3072 | [8,8,1,12,0] | 2.29 | 3795.3159 | 6 | 1238788 |
|  | 0.39 | 3875.2832 | [8,8,1,13,0] | 2.71 | 3875.2727 | 7 | 12924811 |
|  | 0.92 | 3955.2406 | [8,8,1,14,0] | 2.81 | 3955.2295 | 7 | 85752109 |
|  | 0.19 | 4031.2850 | [8,8,1,14,1] | 2.03 | 4031.2932 | 2 | 5565112 |
|  | 0.14 | 3482.2037 | [9,9,1,1,1] | 2.68 | 3482.1944 | 3 | 40939 |
|  |  |  |  |  |  |  |  |
| Replicate 3 | 0.19 | 3795.3006 | [8,8,1,12,0] | 4.04 | 3795.3159 | 6 | 1321287 |
|  | 0.55 | 3875.2801 | [8,8,1,13,0] | 1.91 | 3875.2727 | 6 | 13338628 |
|  | 0.92 | 3955.2375 | [8,8,1,14,0] | 2.01 | 3955.2295 | 7 | 91556509 |
|  | 0.32 | 4014.2597 | [8,8,1,14,1] | 1.72 | 4014.2666 | 5 | 5084741 |
|  | 0.16 | 3482.1959 | [9,9,1,1,1] | 0.43 | 3482.1944 | 2 | 57737 |
