# Supplementary material for: GlycCompSoft: Software for Automated Comparison of Low Molecular Weight Heparins Using Top-Down LC/MS Data
Source: PLoS One. 2016 Dec 12;11(12):e0167727. doi: 10.1371/journal.pone.0167727 (PMC5152843; doi:10.1371/journal.pone.0167727)
Supplement: S2 Table — Components are given out as [HexA, GlcN, PNP = 1, SO3, Ac], and results in red were confirmed as false positive results after manually interpretation. (DOCX) [file pone.0167727.s008.docx]

S2 Table

| DataFrom | Score | MW | Compound Key | PPM Error | Theoretical MW | NumCharges | Total Volume |
| --- | --- | --- | --- | --- | --- | --- | --- |
| Replicate 1 | 0.18 | 3989.2840 | [8,8,1,14,0] | 0.34 | 3989.2826 | 1 | 668826 |
|  | 0.17 | 4434.9659 | [8,8,1,20,0] | 1.02 | 4434.9704 | 6 | 641929 |
|  | 0.17 | 4514.9332 | [8,8,1,21,0] | 1.32 | 4514.9272 | 6 | 2212394 |
|  | 0.14 | 4659.1131 | [8,8,1,21,1] | 3.44 | 4659.0971 | 1 | 1382 |
|  | 0.60 | 4594.8913 | [8,8,1,22,0] | 1.59 | 4594.8840 | 8 | 17739238 |
|  | 0.16 | 4773.1126 | [8,8,1,22,1] | 1.18 | 4773.1070 | 3 | 3732677 |
|  | 0.14 | 5268.5989 | [8,8,1,30,0] | 1.38 | 5268.5916 | 1 | 458 |
|  | 0.14 | 5296.2155 | [9,9,1,24,0] | 0.31 | 5296.2171 | 1 | 444 |
|  |  |  |  |  |  |  |  |
| Replicate 2 | 0.15 | 4006.3090 | [8,8,1,14,0] | 0.04 | 4006.3092 | 1 | 14127 |
|  | 0.15 | 4434.9571 | [8,8,1,20,0] | 3.00 | 4434.9704 | 6 | 531513 |
|  | 0.17 | 4685.2120 | [8,8,1,21,0] | 4.11 | 4685.1927 | 2 | 3758087 |
|  | 0.13 | 4625.0664 | [8,8,1,21,1] | 4.84 | 4625.0440 | 1 | 3206 |
|  | 0.58 | 4594.8923 | [8,8,1,22,0] | 1.81 | 4594.8840 | 8 | 20140278 |
|  | 0.16 | 4773.1217 | [8,8,1,22,1] | 3.09 | 4773.1070 | 2 | 3490949 |
|  | 0.13 | 5285.6111 | [8,8,1,30,0] | 1.34 | 5285.6182 | 1 | 3357 |
|  | 0.13 | 5245.1453 | [9,9,1,24,0] | 1.49 | 5245.1375 | 1 | 565 |
|  |  |  |  |  |  |  |  |
| Replicate 3 | 0.13 | 3989.2854 | [8,8,1,14,0] | 0.70 | 3989.2826 | 1 | 3280 |
|  | 0.16 | 4434.9596 | [8,8,1,20,0] | 2.44 | 4434.9704 | 6 | 578756 |
|  | 0.17 | 4514.9305 | [8,8,1,21,0] | 0.73 | 4514.9272 | 6 | 3122377 |
|  | 0.12 | 4573.9685 | [8,8,1,21,1] | 0.92 | 4573.9643 | 2 | 5242 |
|  | 0.53 | 4765.1635 | [8,8,1,22,0] | 2.93 | 4765.1495 | 8 | 18481026 |
|  | 0.15 | 4756.0944 | [8,8,1,22,1] | 2.93 | 4756.0804 | 2 | 2660006 |
|  | 0.12 | 5370.7710 | [8,8,1,30,0] | 3.73 | 5370.7509 | 1 | 2322 |
|  | 0.12 | 5177.0567 | [9,9,1,24,0] | 4.91 | 5177.0313 | 1 | 1726 |
