# Supplementary material for: GlycCompSoft: Software for Automated Comparison of Low Molecular Weight Heparins Using Top-Down LC/MS Data
Source: PLoS One. 2016 Dec 12;11(12):e0167727. doi: 10.1371/journal.pone.0167727 (PMC5152843; doi:10.1371/journal.pone.0167727)
Supplement: S3 Table — Components are given out as [HexA, GlcN, PNP = 1, SO3, Ac], and results in red were confirmed as false positive results after manually interpretation. (DOCX) [file pone.0167727.s009.docx]

S3 Table

| DataFrom | Score | MW | Compound Key | PPM Error | Theoretical MW | NumCharges | Total Volume |
| --- | --- | --- | --- | --- | --- | --- | --- |
| Replicate 1 | 0.25 | 3989.2816 | [8,8,1,14,0] | 0.25 | 3989.2826 | 6 | 411485 |
|  | 0.18 | 4782.1742 | [8,8,1,22,0] | 0.39 | 4782.1761 | 2 | 259136 |
|  | 0.35 | 4372.2910 | [9,9,1,15,0] | 0.87 | 4372.2872 | 8 | 4086156 |
|  | 0.12 | 4431.3339 | [9,9,1,15,1] | 2.16 | 4431.3243 | 1 | 18483 |
|  | 0.89 | 4520.3718 | [9,9,1,16,0] | 4.77 | 4520.3502 | 4 | 41617630 |
|  | 0.24 | 4579.3687 | [9,9,1,16,1] | 4.08 | 4579.3874 | 1 | 1760534 |
|  |  |  |  |  |  |  |  |
| Replicate 2 | 0.16 | 3989.2843 | [8,8,1,14,0] | 0.42 | 3989.2826 | 3 | 315999 |
|  | 0.14 | 4782.1755 | [8,8,1,22,0] | 0.12 | 4782.1761 | 1 | 38180 |
|  | 0.29 | 4372.2893 | [9,9,1,15,0] | 0.48 | 4372.2872 | 7 | 4335111 |
|  | 0.14 | 4431.3302 | [9,9,1,15,1] | 1.32 | 4431.3243 | 1 | 31641 |
|  | 0.89 | 4520.3722 | [9,9,1,16,0] | 4.86 | 4520.3502 | 4 | 39076509 |
|  | 0.16 | 4545.3212 | [9,9,1,16,1] | 2.87 | 4545.3343 | 1 | 1596571 |
|  |  |  |  |  |  |  |  |
| Replicate 3 | 0.39 | 3955.2273 | [8,8,1,14,0] | 0.57 | 3955.2295 | 6 | 490312 |
|  | 0.13 | 4594.8854 | [8,8,1,22,0] | 0.29 | 4594.8840 | 1 | 4761 |
|  | 0.51 | 4372.2892 | [9,9,1,15,0] | 0.45 | 4372.2872 | 7 | 4052547 |
|  | 0.14 | 4431.3125 | [9,9,1,15,1] | 2.67 | 4431.3243 | 1 | 54240 |
|  | 0.94 | 4520.3717 | [9,9,1,16,0] | 4.76 | 4520.3502 | 4 | 41314376 |
|  | 0.29 | 4545.3300 | [9,9,1,16,1] | 0.94 | 4545.3343 | 4 | 1704927 |
