# Supplementary material for: GlycCompSoft: Software for Automated Comparison of Low Molecular Weight Heparins Using Top-Down LC/MS Data
Source: PLoS One. 2016 Dec 12;11(12):e0167727. doi: 10.1371/journal.pone.0167727 (PMC5152843; doi:10.1371/journal.pone.0167727)
Supplement: S4 Table — Components are given out as [HexA, GlcN, PNP = 1, SO3, Ac], and results in red were is confirmed as false positive results after manually interpretation. (DOCX) [file pone.0167727.s010.docx]

S4 Table

| DataFrom | Score | MW | Compound Key | PPM Error | Theoretical MW | NumCharges | Total Volume |
| --- | --- | --- | --- | --- | --- | --- | --- |
| Replicate 1 | 0.14 | 4731.0948 | [8,8,1,22,0] | 0.35 | 4731.0964 | 2 | 121596 |
|  | 0.14 | 3595.9691 | [8,8,1,6,1] | 3.28 | 3595.9573 | 1 | 375 |
|  | 0.19 | 5091.8954 | [9,9,1,24,0] | 0.61 | 5091.8986 | 5 | 680683 |
|  | 0.51 | 5376.1808 | [9,9,1,25,0] | 1.28 | 5376.1740 | 4 | 8930501 |
|  | 0.14 | 5316.0187 | [9,9,1,25,1] | 1.22 | 5316.0252 | 2 | 127367 |
|  |  |  |  |  |  |  |  |
| Replicate 2 | 0.16 | 4765.1480 | [8,8,1,22,0] | 0.32 | 4765.1495 | 2 | 88139 |
|  | 0.12 | 3459.7410 | [8,8,1,6,1] | 1.11 | 3459.7449 | 1 | 357 |
|  | 0.17 | 5091.8956 | [9,9,1,24,0] | 0.59 | 5091.8986 | 5 | 1455128 |
|  | 0.36 | 5376.1794 | [9,9,1,25,0] | 1.01 | 5376.1740 | 4 | 7721203 |
|  | 0.14 | 5350.0597 | [9,9,1,25,1] | 3.48 | 5350.0783 | 3 | 226158 |
|  |  |  |  |  |  |  |  |
| Replicate 3 | 0.14 | 4594.8826 | [8,8,1,22,0] | 0.31 | 4594.8840 | 2 | 54087 |
|  | 0.13 | 3357.5691 | [8,8,1,6,1] | 4.90 | 3357.5856 | 1 | 506 |
|  | 0.18 | 5091.8952 | [9,9,1,24,0] | 0.67 | 5091.8986 | 4 | 863846 |
|  | 0.36 | 5342.1420 | [9,9,1,25,0] | 3.95 | 5342.1209 | 4 | 9688604 |
|  | 0.14 | 5418.1815 | [9,9,1,25,1] | 0.56 | 5418.1845 | 2 | 27302 |
