# Supplementary material for: GlycCompSoft: Software for Automated Comparison of Low Molecular Weight Heparins Using Top-Down LC/MS Data
Source: PLoS One. 2016 Dec 12;11(12):e0167727. doi: 10.1371/journal.pone.0167727 (PMC5152843; doi:10.1371/journal.pone.0167727)
Supplement: S5 Table — After matching the experimental data with the hypothesis generated by GlycoReSoft, the matching results were further filtrated by GlycoCompSoft using all-presence-principle. The table was outputted as the final matching result. (Components are given out as [ΔHexA = 1, HexA, GlcN, Ac, SO3]). (DOCX) [file pone.0167727.s011.docx]

S5 Table

| Sample names | Score | MW | Compound Key | PPM Error | Theoretical MW | NumCharges | Total Volume |
| --- | --- | --- | --- | --- | --- | --- | --- |
| Generic enoxaparin 1 | 0.28 | 1491.0441 | [1,2,3,0,6] | 0.35 | 1491.0436 | 3 | 51382459 |
| Generic enoxaparin 2 | 0.23 | 1491.0424 | [1,2,3,0,6] | 0.77 | 1491.0436 | 3 | 12058682 |
| Generic enoxaparin 3 | 0.32 | 1491.0444 | [1,2,3,0,6] | 0.54 | 1491.0436 | 3 | 27121143 |
| Generic enoxaparin 1 | 0.32 | 1571.0023 | [1,2,3,0,7] | 1.24 | 1571.0004 | 3 | 171105885 |
| Generic enoxaparin 2 | 0.33 | 1571.0013 | [1,2,3,0,7] | 0.59 | 1571.0004 | 3 | 47421406 |
| Generic enoxaparin 3 | 0.38 | 1571.0029 | [1,2,3,0,7] | 1.60 | 1571.0004 | 2 | 86256827 |
| Generic enoxaparin 1 | 0.49 | 1650.9601 | [1,2,3,0,8] | 1.74 | 1650.9572 | 4 | 373376460 |
| Generic enoxaparin 2 | 0.51 | 1650.9598 | [1,2,3,0,8] | 1.55 | 1650.9572 | 4 | 113654936 |
| Generic enoxaparin 3 | 0.62 | 1650.9605 | [1,2,3,0,8] | 1.97 | 1650.9572 | 4 | 175839962 |
| Generic enoxaparin 1 | 0.43 | 1730.9173 | [1,2,3,0,9] | 1.88 | 1730.9140 | 3 | 299887535 |
| Generic enoxaparin 2 | 0.56 | 1730.9169 | [1,2,3,0,9] | 1.65 | 1730.9140 | 4 | 99291709 |
| Generic enoxaparin 3 | 0.61 | 1730.9175 | [1,2,3,0,9] | 2.02 | 1730.9140 | 4 | 130746181 |
